# Clinical efficacy of ultrasound guided erector spinae plane block in patients undergoing microwave ablation

**DOI:** 10.15537/smj.2022.43.9.20220245

**Published:** 2022-09

**Authors:** Özlem Öz Gergin, Sibel Seçkin Pehlivan, İbrahim Erkan, Adnan Bayram, Recep Aksu, Cihangir Biçer, Karamehmet Yıldız, Güven Kahriman

**Affiliations:** *From the Department of Anesthesiology and Reanimation (Gergin, Pehlivan, Erkan, Bayram, Aksu, Biçer, Yıldız), Medical Faculty, Erciyes University; and Department of Radiology (Kahriman), Medical Faculty, Erciyes University, Kayseri, Turkey.*

**Keywords:** erector spinae plane block, hepatocellular carcinoma, microwave ablation, pain, ultrasonography guidance

## Abstract

**Objectives:**

To compare the effect of pre-emptive erector spinae plane block (ESPB) applied before the procedure on opioid consumption during the procedure and analgesic demand and opioid consumption after the procedure.

**Methods:**

American Society of Anesthesiologists Physical Status Classification (ASA) I-II, 30 patients, with liver tumor and planned for microwave ablation (MWA) treatment were included in the interventional radiology clinic, Erciyes University, Kayseri, Turkey, Turkey between 2021 and 2022. Patients were randomized either to the ESPB or control group. Ultrasound-guided ESPB block with 20 mL of 0.25% bupivacaine was performed preoperatively in the ESPB group patients, and the patients who was not performed the ESPB the control group. All the patients were administered 1 µg/kg fentanyl, 1-2 mg/kg propofol, and 1 mg/kg ketamine for sedation during the MWA procedure after standard monitoring. Total opioid consumption and numeric rating scale (NRS) scores for pain were recorded at 0, 20, 40, and 60 minutes, and at 2, 4, 6, 12, and 24 hours after the procedure.

**Results:**

Total opioid consumption and total opioid amount during the procedure were statistically significantly lower in the ESPB group (*p*<0.001). Although all of the patients in the control group needed additional fentanyl throughout the procedure, only 5 patients in the ESPB group needed additional fentanyl (*p*<0.001). Post-procedure NRS score values were significantly lower in the ESPB group at 40 minutes, 60 minutes and 4 hours (*p*<0.05). Numeric rating scale values at other times were statistically similar (*p*>0.05)

**Conclusion:**

This study showed that ESPB provided effective preemptive analgesia during MWA procedures.

Hepatocellular carcinoma (HCC) is one of the most common tumors in the world. Most HCC patients are not eligible for surgical resection because of insufficient liver function reserve.^1^ Palliative treatment options for unresectable liver tumors include radiofrequency ablation (RF), microwave ablation (MWA), laser, cryotherapy and high-intensity focused ultrasound (HIFU).^2^

Although the ablation procedure is performed with appropriate sedation techniques, the pain felt during the procedure is unpredictable and subjective. It is hypothesized that tumors in the subcapsular region adjacent to the parietal peritoneum or central tumors in contact with large blood vessels may cause severe pain during treatment with RF ablation.^3,4^ After RF ablation treatment for superficial lesions located adjacent to the diaphragm, pain occurs within a few days following the procedure, although it is not common.^5^ The MWA method, which can reach larger ablation areas in shorter times at higher temperatures, increases the success rate in treating liver tumors with successful pain management during the procedure.^6^ While tumor ablation can be performed under local or general anesthesia, conscious sedation practices accompanied by monitored anesthesia care (MAC) have recently also taken their place in clinical practice.^7,8^ Although opioids have adverse effects such as respiratory depression, nausea and vomiting, they are still widely used in ablation treatments, as their doses can be adjusted according to the severity of pain and are very effective in providing analgesia.^8,9^

Erector spinae plane block (ESPB) was first defined and applied by Forero^10^ in 2016 as an ultrasound-guided interfascial plane block for the treatment of thoracic neuropathic pain. Erector spinae plane block is based on the principle of blocking the dorsal and ventral roots of the thoracic and abdominal spinal nerves. Ultrasound-guided ESPB has been widely used to treat acute and chronic pain in recent years.^11-13^ This study primarily aimed to compare the effect of preemptive ESPB applied before the procedure on fentanyl consumption during the procedure and analgesic demand and consumption after the procedure. Our secondary aim was to investigate the postoperative pain levels of our patients and their effects on radiologist satisfaction.

## Methods

This study was planned patients with liver tumors who were examined underwent ablation in the interventional radiology clinic at Erciyes University, Kayseri, Turkey between 2021 and 2022. This study was approved by the Erciyes University Faculty of Medicine Clinical Research Ethics Committee with protocol number 2021/346 dated 05.05.2021 and complied with the principles of the Declaration of Helsinki. The protocol of the study was registered at Clinical Trials.gov (NCT05009550) before study initiation and publication. The study was performed as prospective, randomized and single-centered. Thirty volunteer patients aged 18-65 years, within the American Society of Anesthesiologists Physical Status Classification (ASA) I-II risk group, diagnosed with a liver tumor and planned for MWA treatment, who were admitted to Erciyes University Faculty of Medicine Interventional Radiology Clinic, were included in the study by obtaining informed consent. Risk scale of ASA III-V, patients with concomitant severe cardiac, respiratory, allergy history, coagulopathy, patients receiving opioid therapy for chronic pain, patients with low cognitive functions incapable of evaluating the numerical pain scale (NRS) score, morbid obesity and patient requests not to be included were determined as the exclusion criteria.

The patients were randomly assigned to the ESPB (n=15) and control (n=15) groups. Randomization was achieved using a computer‐based list before the procedure. Demographic data of the patients (age, height, weight, gender, comorbidity, ASA scores), post-procedure analgesic use, first rescue analgesic time and NRS were recorded. Standard monitoring (electrocardiogram, pulse oximetry, and non-invasive blood pressure) was applied to the patients who were taken to the MWA procedure room, and vascular access was established. A 2 L/min oxygen support was administered by nasal cannula. All patients were informed regarding the postoperative pain assessment score NRS.

### Erector spinae plane block technique

An experienced anesthesiologist performed all ESPB procedures. ESPB was performed with USG (Sonosite M Turbo®, Fujifilm Inc., USA) preemptively 30 minutes (mins) before the procedure in patients who accepted ESPB. The patients were laid in the prone position, and after the skin was cleaned, a linear 38-mm, high frequency 10-15 MHz transducer linear USG probe was placed 2-3 cm lateral to the T8 vertebral spinous process in the paramedian sagittal plane. Local anesthesia was achieved by infiltrating 1 mL of 2% lidocaine into the skin and subcutaneous tissue at the needle insertion sites. In the in-plane, a 22 gauge 80 mm stimuplex needle (B. Braun Medical Inc., Bethlehem, PA, USA) was inserted in the cranial-caudal direction until it came into contact with the T8 transverse process. A 20 mL of 0.25% bupivacaine hydrochloride (Bustesin, Vem Drug Company, Istanbul, Turkey) (prepared by adding 10 mL of 0.5% bupivacaine + 10 mL of SF) was administered. The distribution of local anesthetic was confirmed by observing in the plane between the transverse process of the T8 vertebra and the erector spinae muscle, and the procedure was terminated. At the 30th minute after the procedure, the degree of sensory blockade of the patients was evaluated on a 3-point scale (0=normal sensation; 1=feels the needle slightly; 2=no sensation) with the pinprick test. As a result of the sensorial block evaluation, scores of 1 and above on 3-point scales were accepted as sufficient block, and the MWA procedure was initiated. Patients in the control group and ESPB group were administered 1 µg/kg fentanyl, 1-2 mg/kg propofol and 1 mg/kg ketamine for sedation during the MWA procedure after standard monitoring.

### Microwave ablation (MWA) protocol

A 15-gauge liquid-cooled antenna and a 2.45 GHz generator with a power of 120 W (Solero microwave tissue ablation system; Angiodynamics, USA) were used for the microwave ablation procedure. The duration of the procedure varied depending on the size of the nodule to be ablated.

Sedation of the patients during the MWA procedure was evaluated with the Observer’s Assessment of Alertness/Sedation Scale (OAASS) (5=response easily to name spoken in normal tone; 4=lethargic response to name spoken in normal tone; 3=responds only after name is called loudly or repeatedly; 2=responds only after mild shaking; 1=does not respond to mild shaking). According to the observer evaluation on the alertness/sedation scale, 3 or more was considered adequate sedation. At this level of sedation, the patients seemed relaxed and could maintain their spontaneous breathing. During the MWA procedure, it was aimed to achieve the targeted depth of anesthesia and the need for analgesia by paying attention to 20% or more changes in the initial values of heart rate and blood pressure (repeated as 50% of the initial dose of the analgesic agent used). Bradycardia were treated with 0.01 mg kg-1 atropine IV and hypotension were treated with 0.1 mg kg-1 ephedrine IV. The satisfaction of the interventional radiologist during the procedure was evaluated on a satisfaction scale (1=not at all satisfied; 2=not satisfied; 3=reasonable; 4=satisfied; 5=highly satisfied). Before and after the MWA procedure, patients in both the control and ESPB groups were asked to rate their pain from 0 to 10 with NRS to define their pain intensity, wherein 0 means no pain, and 10 at the other end of the scale represents unbearable pain. NRS values were evaluated by an anesthesiologist blinded to the study who did not know to which study group the patient belonged. Patients in the recovery unit were evaluated with the modified aldrete score, and if the score was 9 and above, the patient was transported to the ward safely.^9^

Hemodynamic data, NRS pain scores, whether there was a need for rescue analgesics, time of first analgesic use, and nausea, vomiting, and other complications of the patients in the recovery unit at 0, 20, 40 and 60 mins and at 2, 4, 6, 12 and 24 hours after the procedure were recorded. The total amount of fentanyl and propofol consumed during the MWA procedure, the need for additional fentanyl and the use of analgesics after the procedure, the time of first analgesic use, and the amount of analgesic used were also noted. Patient follow-up in the postoperative period was performed by an anesthetist who was blinded to the study and did not know which study group the patient belonged to. During the follow-up, paracetamol 1 g IV (Partemol® Vem Istanbul, Turkey) was administered as a rescue analgesic in case of NRS>4, and contramal 30 mg IV (Tramosel®, Haver, Istanbul, Turkey) was applied if this was insufficient.

According to the power analysis performed before the study, to reveal the intraoperative fentanyl consumption difference between the 2 groups, the number of patients in the study was calculated as at least 15 in each group, with a total of 30, with type 1 error of 5%, effect size=2.424, and statistical power=99.9%.

### Statistical analysis

Data were evaluated using the Statistical Package for the Social Sciences, version 26 (IBM Corp., Armonk, New York, USA) statistical package program. Descriptive statistics were presented as the number of units, percent, mean ± standard deviation, median, minimum, maximum, and interquartile range (IQR) values. The normal distribution of the data of numerical variables was evaluated with the Shapiro Wilk test of normality. Normally distributed variables between groups were compared with independent samples t-test. Mann-Whitney U test was used to compare numerical variables that did not show normal distribution according to the groups. The exact method of the Pearson Chi-square test compared the study groups with categorical variables. If the Chi-square test result was statistically significant, subgroup analyses were performed with Bonferroni-corrected 2-proportion z-test. Linear mixed models were used since there were missing values in the comparison of the heart rate, systolic arterial blood pressure (SABP) diastolic arterial blood pressure, and oxygen saturaiton values of the groups according to the measurement times. Comparison of NRS scores according to measurement times was performed with 2-way repeated measures analysis of variance. Bonferroni correction was applied for multiple comparisons. A value of *p*<0.05 was considered statistically significant.

## Results

When the 2 groups were compared in terms of age, gender, ASA score distribution, subcapsular/intraparenchymal location, lesion size and procedure time, there was no statistically significant difference in the distribution of values (*p*>0.05) (Table 1). Heart rate was significantly higher in the control group at the 5th min. While SABP values at 0 min were significantly higher in the ESPB group, they were statistically higher at the 5th minute in the control group (*p*<0.05) (Table 1). Oxygen saturation values revealed a statistically similar distribution at 0, 5, 10, and 15 min (*p*>0.05) (Table 2).

**Table 1:** - Comparison of descriptive characteristics by groups.

Variables	Group control (n=15)	Group ESPB (n=15)	*P*-value
***Age (years)***			
Mean±sd	64.2±12.6	63.8±8.7	0.934
Min-max	34-77	47-76	
***Gender, n (%)***			
Female	6 (40.0)	8 (53.3)	0.715
Male	9 (60.0)	7 (46.7)	
***ASA, n***			
Grade I	3	2	0.999
Grade II	12	13	
***Localization, n (%)***			0.143
Subcapsular	10 (66.7)	5 (33.3)	0.143
Intraparenchymal	5 (33.3)	10 (66.7)	
***Lesion size***			
Mean±sd	4.1±2.5	2.7±1.1	0.058
Min-max	(1.80-12.00)	(1.20-5.50)	
***Duration of procedure***			
Mean±sd	9.8±4.24	11.6±3.61	0.267
Min-max	4.0-15.0	5.0-15.0	

Values are presented as number and percentage (%). ESPB: erector spinae plane block, ASA: American Society of Anesthesiology, Sd: standard deviation

**Table 2:** - Comparison of heart rate, systolic arterial blood pressure, diastolic arterial blood pressure, and oxygen saturation values by groups (N=25).

Parameters	Groups		Test statistics†	
	Group control (mean±sd)	Group ESPB (mean±sd)	F	*P*-value
***Heart rate***				
0th min of the intervention	81.93±2.67^a^	79.33±2.67	0.473	0.497
5th min of the intervention	95.00±1.51^b^	74.07±1.51	96.568	<0.001
10th min of the intervention	81.88±2.80^a^	79.52±2.46	0.402	0.533
15th min of the intervention	74.73±3.76^a^	77.18±3.18	0.247	0.628
Test statistics‡	F=31.816; *p*<0.001	F=1.981; *p*=0.147		
***Systolic arterial blood pressure***				
0th min of the intervention	131.67±6.21^a^	152.73±6.21^a^	5.754	0.023
5th min of the intervention	150.53±2.63^b^	128.47±2.63^b^	35.267	<0.001
10th min of the intervention	144.26±7.77^ab^	153.99±6.99^a^	0.866	0.361
15th min of the intervention	151.54±8.28^ab^	149.32±7.40^a^	0.040	0.843
Test statistics‡	F=4.067; *p*=0.023	F=10.554; *p*<0.001		
***Diastolic arterial blood pressure***				
0th min of the intervention	79.73±3.34^a^	83.13±3.34^a^	0.519	0.477
5th min of the intervention	95.33±1.91^b^	75.40±1.91^b^	54.575	<0.001
10th min of the intervention	88.83±3.51^ab^	88.09±3.22^ab^	0.024	0.878
15th min of the intervention	90.14±4.78^ab^	78.85±4.23^ab^	3.127	0.092
Test statistics‡	F=12.444; *p*<0.001	F=17.685; *p*<0.001		
***Oxygen saturation***				
0th min of the intervention	97.13±0.49^a^	96.20±0.49^a^	1.791	0.192
5th min of the intervention	97.27±0.38^a^	97.67±0.38^ab^	0.558	0.461
10th min of the intervention	97.54±0.38^a^	97.88±0.33^b^	0.475	0.497
15th min of the intervention	97.87±0.38^b^	97.77±0.34^b^	0.041	0.840
Test statistics‡	F=7.028; *p*=0.002	F=3.202; *p*=0.048		

*Linear mixed models, †Intergroup comparisons on each measure, ‡Comparisons between measurements in each group a and b superscripts indicate within-group measurement differences. Measurements with the same letters were statistically similar. F: Fisher exact test, min: minutes

Total opioid consumption and total opioid amount during the procedure were statistically significantly lower in the ESPB group (*p*<0.001). Although all of the patients in the control group needed additional fentanyl throughout the procedure, only 5 patients in the ESPB group needed additional fentanyl (*p*<0.001) (Table 3). The total amount of propofol consumed during the procedure, the analgesic consumption after the procedure, and the time of first analgesic use after the procedure were statistically similar in the groups (*p*>0.05) (Table 3). When both groups were compared in terms of pre-procedural NRS score values, the distribution of values was statistically similar (*p*>0.05) (Table 4). Post-procedure NRS score values were significantly lower in the ESPB group at 40 and 60 mins, and at 4 hours (*p*<0.05) (Table 4). NRS values at other times were statistically similar (*p*>0.05) (Table 4).

**Table 3:** - Analgesic consumption during and after the procedure.

Variables	Group control (n=15)	Group ESPB (n=15)	*P*-value
Total opioid consumption (fentanyl in mcg), M (IQR)	100.0 (50.0)	25.0 (25.0)	<0.001^†^
Total opioid amount (fentanyl in mcg/kg/min), M (IQR)	0.1 (0.06)	0.0 (0.0)	<0.001^†^
***Additional fentanyl consumption throughout the procedure, n (%)***			
Not performed	0 (0.0)	10 (66.7)	<0.001^‡^
Performed	15 (100.0)	5 (33.3)	
Total Propofol amount (Propofol in mg), M (IQR)	35.0 (10.0)	40.0 (15.0)	0.325^†^
***Analgesic consumption after the procedure, n (%)***			
Yes	11 (7.3)	5 (33.3)	0.066^‡^
No	4 (26.7)	10 (66.7)	
Time of first analgesic use after the procedure (hours), M (IQR)	2.0 (3.0)	4.3 (12.8)	0.851^†^

Values are presented as number and percentages (%). M: median, IQR: interquartile range, †Mann-Whitney U test, ‡Fisher exact test, ESPB: erector spinae plane block

**Table 4:** - Distribution of NRS scores by groups.*

NRS score (time)	Group control	Group ESPB	*P*-value^†^
	mean±sd	mean±sd	
Pre-procedure	0.60±1.30	0.67±1.23	0.886
Post-procedure 0th min	1.20±1.08	1.00±1.13	0.625
20th minute	2.27±1.39	1.60±1.24	0.176
40th minute	2.73±1.03	1.60±1.18	0.009
60th minute	3.00±1.36	1.40±0.99	0.001
2nd hour	3.20±1.70	2.00±0.85	0.021
4th hour	2.87±0.74	1.87±0.74	0.001
6th hour	2.60±0.99	2.20±1.01	0.283
12th hour	1.93±1.03	1.93±0.88	>0.999
24th hour	1.20±0.56	1.80±0.86	0.032

Sd: standard deviation, NRS : Numerical Pain Scale, *: Two-way repeated measures analysis of variance, †: *p*-values with Bonferroni correction

The presence of postoperative complications was statistically similar between the groups (*p*<0.999). Only one person from the control group developed respiratory depression. Radiology doctor satisfaction scores were statistically different between ESPB and control groups. While the number of patients who expressed that they were not satisfied with the radiologist was statistically higher in the control group, the number of patients whose satisfaction they expressed as excellent was statistically higher in the ESPB group (*p*<0.001) (Table 5).

**Table 5:** - Comparison of post-procedure complication rates and radiology doctor satisfaction levels between groups (N=15).

Variables	Group control	Group ESPB	*P*-value
***Postoperative complications, n (%)***			
Yes	1 (6.7)	0 (0.0)	0.999
No	14 (93.3)	15 (100.0)	
***Radiology doctor satisfaction score***			
Not satisfied at all	1 (6.7)^a^	0 (0.0)^a^	<0.001
Not satisfied	7 (46.7)^a^	0 (0.0)b	
Reasonable	6 (40.0)^a^	2 (13.3)^a^	
Satisfied	1 (6.7)^a^	4 (26.7)^a^	
Excellent	0 (0.0)^a^	9 (60.0)^b^	

Superscripts a and b indicate the difference between groups in each category. Groups with the same letters in each category were statistically similar. ESPB: erector spinae plane block

## Discussion

This study observed that ESPB administered preemptively as part of multimodal analgesia in the MWA procedure reduced total opioid consumption and the need for additional analgesics both in the perioperative and postoperative periods. Radiofrequency ablation and MWA methods have been reported to be quite effective in treating liver tumors, either in combination with surgery or as a stand-alone application.^3^ Microwave thermal ablation methods have been developed as an alternative to traditional radiofrequency ablation methods since they can produce larger and hotter ablations.^6,7^

Pain, pleural effusion, development of perihepatic fluid or blood collections are common complications after ablation procedures.^14^ It is generally accepted that the liver parenchyma is insensitive to pain. Pain during ablation procedures is generally associated with parietal peritoneal irritation in the presence of central tumors adjacent to large vessels, or the presence of a superficial tumor.^5,15^

Understanding the general characteristics of the tumor, such as the size and localization of the tumor, and the factors associated with pain during the procedure will help predict the analgesic requirements during the procedure.^3,15^ General anesthesia, sedation or local anesthesia are preferred during tumor ablation. When general anesthesia is preferred, muscle relaxants ensure precise placement of the applicator and immobility of the patient throughout the procedure. Performing ablation procedures with deep or conscious sedation can save time by shortening the start of the procedure and providing faster recovery after the procedure is completed, as well as protecting the patient from potential risks associated with general anesthesia.^7^ Opioids are widely used analgesics during tumor ablation therapy, but they have disadvantages such as respiratory depression, bradycardia, hypotension, nausea-vomiting and apnea.^8,9,15,16^

Erector spinae plane block is increasingly used in many different regions and different pain indications for selective multidermatomal sensory blockade in postoperative or neuropathic pain.^17-21^ ESPB, which was first defined for the treatment of thoracic neuropathic pain by Forero et al,^10^ is one of the regional anesthesia methods with proven effectiveness to reduce intraoperative and postoperative opioid consumption as well as to provide postoperative analgesia.

When ESPB is administered at the T5-6 level in thoracic surgeries and the T7-9 level in abdominal surgeries, anesthesia and analgesia can be provided in the area to be treated. It was reported that the local anesthetic agent injected after ESPB moves in the cephalo-caudal direction with the thoracolumbar fascia and also blocks the sympathetic fibers by spreading to the paravertebral area. Erector spinae plane block can prevent visceral pain in addition to somatic pain.^21,22^ Tulgar et al^23^ reported that bilateral sensory block was achieved after unilateral ESPB was applied at the T9 level. In this case, when this study was planned, it was revealed that ESPB could provide effective analgesia when performed unilaterally. Moreover, before starting the ablation process after ESPB, when the sensory blocks of the patients were evaluated with the pinprick test, the block was accepted to be sufficient according to whether they felt the needle slightly or not.

In many studies investigating the effects of ESPB on acute and chronic pain, when ESP block is used as a part of a multimodal analgesia plan, it was reported in the literature to be an interfascial plane block with a wide range of indications.^12,20,24,25^ Goel et al^26^ reported that intraoperative and postoperative 24-hour total opioid consumption in lumbar spinal fusion surgery was lower in the block group than in the control group. Compared to the block group, NRS scores of the control group were reported to be significantly higher in the first 48 hours after surgery. Krishna et al^27^ reported that the need for fentanyl decreased significantly in the intraoperative and postoperative periods in patients who underwent ESPB in the preoperative period in their randomized controlled study with 106 patients who were going to undergo cardiac surgery. In another study in which thoracic epidural analgesia and ESPB methods were compared in 50 patients who underwent cardiac surgery, they reported the amount of fentanyl used in the ESPB group in the intraoperative and postoperative period was significantly lower.^28^ In this study, the total opioid consumption during the MWA procedure and the total amount of opioids needed together with the post-procedure period was lower in the ESPB group (*p*<0.001). Although all of the patients in the control group needed additional fentanyl throughout the procedure, only 5 patients in the ESPB group needed additional fentanyl (*p*<0.001). The difference in total opioid consumption and additional fentanyl need during the MWA procedure in both groups can be explained in that the local anesthetic injected with ESPB moves along the fascia in the paravertebral, caudal and cephalic directions and provides effective somatic and visceral analgesia in a wide area from the C7-T2 dermatomal level to the L2-3 level.

The literature reported that ESPB provides effective analgesia as a part of multimodal analgesia in the postoperative period.^23,24^ Krishna et al^27^ reported that NRS scores were significantly lower in patients who underwent ESPB compared to the control group (*p*<0.05), and the time of the first analgesia was 10 hours in the ESPB group and 6 hours in the control group. In a similar study,^29^ it was reported that NRS scores were significantly lower at postoperative 15 and 30 mins, and 12 and 24 hours in patients who underwent USG-guided ESPB in laparoscopic cholecystectomy surgery (*p*<0.05). Contrary to these studies, the study of Gürkan et al^30^ with 50 patients undergoing breast surgery and Yörükoğlu et al^31^ in their study of 60 patients undergoing lumbar disc surgery, they found no significant difference in pain scores between patients in the ESPB group and control group. In this study, 40-60 mins and 4-hour NRS score values after the procedure were significantly lower in the ESPB group than the group for whom block was not applied (*p*=0.013, 0.001 and *p*=0.003). It was determined that there was no statistically significant difference between the NRS score values between the groups at other times. After the procedure, analgesic consumption and first analgesic use time were statistically similar in the groups.

Altıparmak et al^29^ reported no significant difference between complication rates in patients who underwent transverse abdominis plane block and ESPB. Similarly, the presence of postoperative complications (such as nausea-vomiting, hypotension, and other complications) was statistically similar in this study (*p*<0.001). This statistical similarity can be explained by the fact that the patients included in the study received prophylaxis treatment for nausea and vomiting along with the chemotherapeutic drugs they used. Although the MWA procedure is performed with USG, successful pain control in patients and good focus of the radiologist prevent the development of many undesirable serious complications. In this study, however, the radiologist’s satisfaction with continuing the MWA procedure without interruption was questioned, and the number of patients who expressed their satisfaction as excellent was statistically higher in the ESP block group.

### Study limitations

The first limitation was that the patients had knowledge on the regional technique applied, therefore the participants could not be completely blinded to the study, since some of the patients who underwent ESPB had previous experience of MWA performed without blocking. Another limitation may be that the patients included in the study received prophylaxis against nausea/vomiting with immunosuppressive drugs, and the effect of ESPB on the incidence of nausea/vomiting could not be evaluated.

In conclusion, we believe that preemptive application of ESPB during MWA procedures has advantages such as reducing perioperative and postoperative analgesic consumption, reducing the need and amount of additional analgesics, reducing minor complications such as nausea and vomiting associated with opioids, and increasing the satisfaction of the radiologist, as well as being an effective method in terms of providing high patient satisfaction by increasing treatment compliance in repetitive sessions by preventing anxiety and psychological trauma that may occur in patients due to the procedure.

## References

[1] Choti MA. Surgical management of hepatocellular carcinoma: resection and ablation. J Vasc Interv Radiol 2002; 13: S197–S203. 12354837 10.1016/s1051-0443(07)61787-4

[2] Narayanan G , Hosein PJ , Arora G , Barbery KJ , Froud T , Livingstone AS , et al. Percutaneous irreversible electroporation for downstaging and control of unresectable pancreatic adenocarcinoma. J Vasc Interv Radiol 2012; 23: 1613–1621. 23177107 10.1016/j.jvir.2012.09.012

[3] Goldberg SN , Charboneau JW , Dodd GD III , Dupuy DE , Gervais DA , Gillams AR , et al. Imageguided tumor ablation: proposal for standardization of terms and reporting criteria. Radiology 2003; 228: 335–345. 12893895 10.1148/radiol.2282021787

[4] Lim HK. Radiofrequency thermal ablation of hepatocellular carcinomas. Korean J Radiol 2000; 1: 175–184. 11752952 10.3348/kjr.2000.1.4.175 PMC2718198

[5] Livraghi T , Solbiati L , Meloni MF , GS Gazelle , Halpern EF , Goldberg SN , et al. Treatment of focal liver tumors with percutaneous radio-frequency ablation: complications encountered in a multicenter study. Radiology 2003; 226: 441–451. 12563138 10.1148/radiol.2262012198

[6] Lubner MG , Brace CL , Hinshaw JL , Lee FTJ. Microwave tumor ablation: mechanism of action, clinical results and devices. J Vasc Interv Radiol 2010; 21: S192–S203. 20656229 10.1016/j.jvir.2010.04.007 PMC3065977

[7] Wells SA , Hinshaw JL , Lubner MG , CL Brace , Lee Jr FT , et al. liver ablation: best practice. Radiol Clin North Am 2015; 53: 933–971. 26321447 10.1016/j.rcl.2015.05.012 PMC6419975

[8] Joung KW , Choi SS , Jang DM , Kong YG , Lee HM , Shim JH , et al. Comparative effects of dexmedetomidine and propofol on US-guided radiofrequency ablation of hepatic neoplasm under monitored anesthesia care: a randomized controlled study. Medicine (Baltimore) 2015; 94: e1349. 26266387 10.1097/MD.0000000000001349 PMC4616670

[9] Tobias JD , Leder M. Procedural sedation: A review of sedative agents, monitoring, and management of complications. Saudi J Anaesth 2011; 5: 395–410. 22144928 10.4103/1658-354X.87270 PMC3227310

[10] Forero M , Adhikary SD , Lopez H , Tsui C , Chin KJ. The erector spinae plane block: a novel analgesic technique in thoracic neuropathic pain. Reg Anesth Pain Med 2016; 41: 621–627. 27501016 10.1097/AAP.0000000000000451

[11] Cassai DA , Bonvicini D , Correale C , Sandei L , Tulgar S , Tonetti T. Erector spinae plane block: a systematic qualitative review. Minerva Anestesiol 2019; 85: 308–319. 30621377 10.23736/S0375-9393.18.13341-4

[12] Kot P , Rodriguez P , Granell M , Cano B , Rovira L , Morales J , et al. The erector spinae plane block: a narrative review. Korean J Anesthesiol 2019; 72: 209–220. 30886130 10.4097/kja.d.19.00012 PMC6547235

[13] Aydın T , Balaban O , Ahiskalioglu A , Alici HA , Acar A. Ultrasound-guided Erector Spinae Plane Block for the Management of Herpes Zoster Pain: Observational Study. Cureus 2019; 11; e5891. 31772861 10.7759/cureus.5891 PMC6837261

[14] Rhim H. Complications of radiofrequency ablation in hepatocellular carcinoma. Abdom Imaging 2005; 30: 409–418. 15688113 10.1007/s00261-004-0255-7

[15] Lee S , Rhim H , Kim YS , Choi D , Lee WJ , Lim HK , et al. Percutaneous radiofrequency ablation of hepatocellular carcinomas: factors related to intraprocedural and postprocedural pain. AJR Am J Roentgenol 2009; 192: 1064–1070. 19304715 10.2214/AJR.08.1350

[16] Peng PW , Sandler AN. A review of the use of fentanyl analgesia in the management of acute pain in adults. Anesthesiology 1999; 90: 576–99. 9952166 10.1097/00000542-199902000-00034

[17] Abdelhamid K , ElHawary H , Turner JP. The use of the erectorspinae plane block to decrease pain and opioid consumption in the emergency department: a literature review. J Emerg Med 2020; 58: 603–609. 32245689 10.1016/j.jemermed.2020.02.022

[18] Tsui BC , Fonseca A , Munshey F , McFadyen G , Caruso TJ. The erector spinae plane (ESP) block: a pooled review of 242 cases. J Clin Anesth 2019; 53: 29–34. 30292068 10.1016/j.jclinane.2018.09.036

[19] Aydin ME , Ahiskalioglu A , Tekin E , Ozkaya F , Ahiskalıoğlu EO , Bayramoğlu A. Relief of refractory renal colic in emergency department: a novel indication for ultrasound guided erector spinae plane block. Am J Emerg Med 2019; 37: 794.e1–794.e3. 10.1016/j.ajem.2018.12.042 30595427

[20] Tekin E , Ahiskalioglu A , Aydin ME , Bayramoglu A , Alici HA. High thoracic ultrasound-guided erector spinae plane block for acute herpes zoster pain management in emergency department. Am J Emerg Med 2019; 37: 375–372. 10.1016/j.ajem.2018.10.028 30340986

[21] Chin KJ , Adhikary S , Sarwani N , Forero M. The analgesic efficacy of pre-operative bilateral erector spinae plane (ESP) blocks in patients having ventral hernia repair. Anaesthesia 2017; 72: 452–460. 28188621 10.1111/anae.13814

[22] Tulgar S , Selvi O , Kapakli MS. Erector spinae plane block for different laparoscopic abdominal surgeries: case series. Case Rep Anesthesiol 2018; 2018: 1–3. 10.1155/2018/3947281 PMC5835299 29670771

[23] Tulgar S , Selvi O , Ahiskalioglu A , Ozer Z. Can unilateral erector spinae plane block result in bilateral sensory blockade? Can J Anaesth 2019; 66: 1001–1002. 31114943 10.1007/s12630-019-01402-y

[24] Forero M , Rajarathinam M , Adhikary SD , Chin KJ. Erector spinae plane block for the management of chronic shoulder pain: a case report. Can J Anaesth 2018; 65: 288–293. 29134518 10.1007/s12630-017-1010-1

[25] Schwartzmann A , Peng P , Maciel MA , Forero M. Mechanism of the erector spinae plane block: insights from a magnetic resonance imaging study. Can J Anaesth 2018; 65: 1165–1166. 30076575 10.1007/s12630-018-1187-y

[26] Goel VK , Chandramohan M , Murugan C , Shetty AP , Subramanian B , Kanna RM , et al. Clinical efficacy of ultrasound guided bilateral erector spinae block for single level lumbar fusion surgery: A prospective, randomized, case-control study. Spine J 2021; 21: 1873–1880. 34171466 10.1016/j.spinee.2021.06.015

[27] Krishna SN , Chauhan S , Bhoi D , Kaushal B , Hasija S , Sangdup T , et al. Bilateral erector spinae plane block for acute post-surgical pain in adult cardiac surgical patients: a randomized controlled trial. J Cardiothorac Vasc Anesth 2019; 33: 368–375. 30055991 10.1053/j.jvca.2018.05.050

[28] Nagaraja PS , Agavendran S , Singh NG , Asai O , Bhavya G , Manjunath N , et al. Comparison of continuous thoracic epidural analgesia with bilateral erector spinae plane block for perioperative pain management in cardiac surgery. Ann Card Anaesth 2018; 21: 323–327. 30052229 10.4103/aca.ACA_16_18 PMC6078032

[29] Altıparmak B , Korkmaz Toker M , Uysal AI , Kuşçu Y , Demirbilek GS. Ultrasound-guided erector spinae plane block versus oblique subcostal transversus abdominis plane block for postoperative analgesia of adult patients undergoing laparoscopic cholecystectomy: Randomized, controlled trial. J Clin Anesth 2019; 57: 31–36. 30851501 10.1016/j.jclinane.2019.03.012

[30] Gürkan Y , Aksu C , Kuş A , Yörükoğlu UH , Kılıç CT. Ultrasound guided erector spinae plane block reduces postoperative opioid consumption following breast surgery: A randomized controlled study. J Clin Anesth 2018; 50: 65–68. 29980005 10.1016/j.jclinane.2018.06.033

[31] Yörükoğlu HU , İçli D , Aksu C , Cesur S , Kuş A , Gürkan Y. Erector spinae block for postoperative pain management in lumbar disc hernia repair. J Anesth 2021; 35: 420–425. 33751203 10.1007/s00540-021-02920-0

